# Leveraging Data to Drive Bundle Compliance and Reduce Sepsis-related Mortality

**DOI:** 10.1097/pq9.0000000000000520

**Published:** 2021-12-16

**Authors:** Adrienne M. Marcinick, Gabriella A. Butler, Andrew R. Buchert, Tracy A. Pasek, Christopher M. Horvat

## Abstract

The Children’s Hospital Association’s Improving Pediatric Sepsis Outcomes (IPSO) collaborative is a multi-center quality improvement (QI) learning collaborative of 61 U.S. children’s hospitals that seeks to improve sepsis outcomes through collaborative learning and reliable implementation of evidence-based interventions in pediatric emergency departments, intensive care units, general care units, and hematology/oncology units. Specifically, IPSO’s goals are to decrease sepsis-attributable mortality and prevent hospital-onset sepsis among children.

The following 10 abstracts represent a select group of projects undertaken by IPSO participating hospitals that were presented at one of three collaborative events in 2020 and 2021. IPSO’s Research Workgroup reviewed all submitted abstracts and selected the top 10 for inclusion in this Supplement

## Introduction:

Before 2018, the hospital did not have an automated mechanism to consistently identify patients at risk for developing sepsis, tools within the electronic medical record to capture the completion of sepsis screening, nor the data pipeline to provide feedback on the performance measures of the sepsis bundle. Without a standardized pathway, clinical decision support, and data infrastructure, a high degree of variability was observed between care teams. The objective was to utilize a data-driven approach to standardize the sepsis screening process and to hardwire the response of care teams, ultimately leading to timely recognition and treatment of sepsis, thereby reducing mortality.^1^

## Methods:

Given the specialization of the patient population, observed practice variability within care teams, and the need for both tools at the point of care as well as data dissemination and feedback, multiple high-yield interventions were developed. To support the identification and treatment of patients at risk for sepsis, the team designed an electronic triggering system and electronic medical record-integrated tools. To drive ongoing improvement and sustainability, they built a new clinical dashboard to measure performance and outcomes, automated daily reports, facilitated staff meetings to share “good catches” and “near misses” associated with individual sepsis events, and targeted rounding and education within the clinical areas.^2^

## Resutls:

**Fig. 1.**
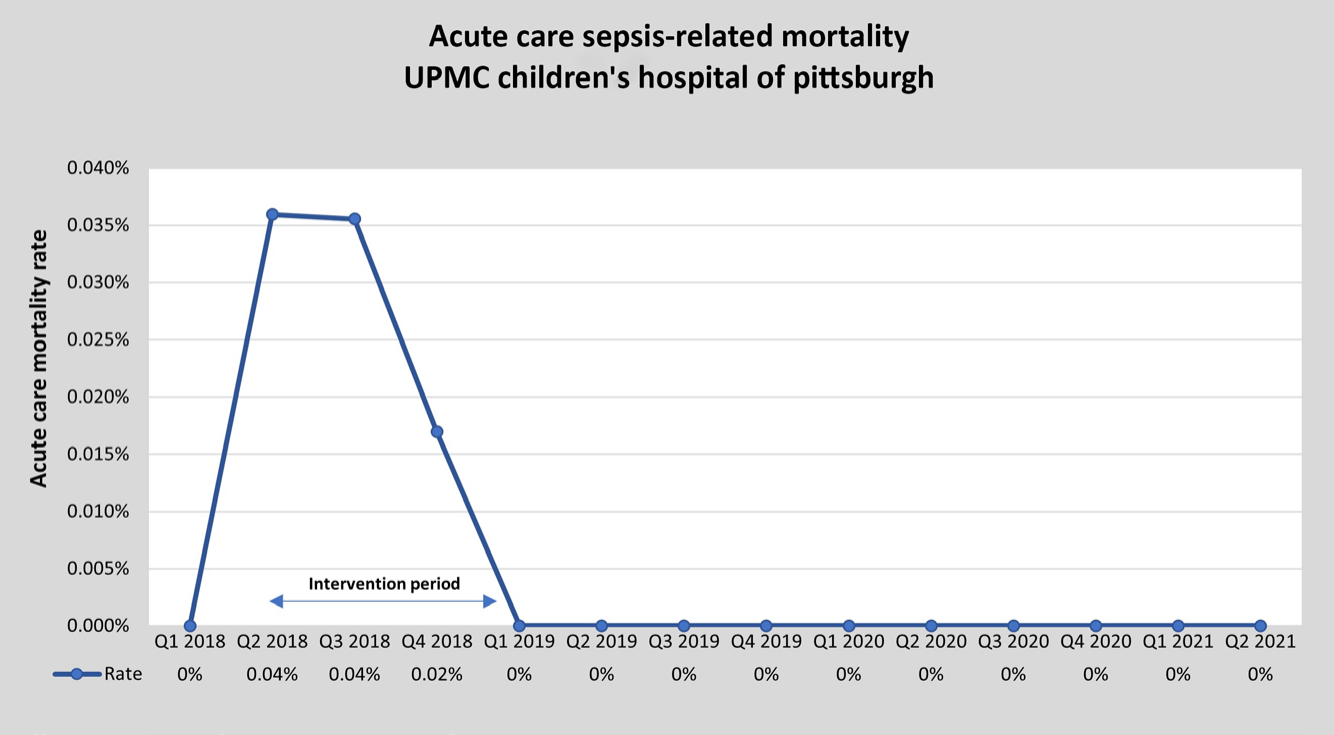
Acute care sepsis-related mortality. Numerator, #acute care deaths related to sepsis; Denominator, #acute care discharges. No formal data collection of acute care sepsis-related mortality before January 2018.

Since the rolling go-live of the acute care sepsis pathway, the hospital has achieved and sustained a 0% sepsis-related mortality rate on acute care units (Fig. 1).

## Conclusion:

Accurate and timely feedback regarding process metrics and sepsis events yielded improved patient outcomes. Sustained reliability of the sepsis pathway with a resultant decrease in sepsis-related mortality was achieved. Daily distribution of automated compliance reports distributed locally and to leadership is critical for buy-in.

## Acknowledgments:

Assistance with the study: Johanna Rosen, MD; Lauren Alessi, MD; Vikram Raghu, MD; Joseph Carcillo, MD; Vikram Raghu, MD; Zachary Aldewereld, MD; Ashley Kimble, MD; Daniel Loeb, MD; Kristina Gaietto, MD; Michael Freedman, MD; Allison Rometo, MD; Neema Shah, MD; Katharina Hayes, MD; James Bowen, MD; Jeffrey Rudolph, MD; Matthew Valente, MD; Priya Marathe, MD; Sylvia Choi, MD; Elizabeth Landsberg, MD; Elizabeth Ferguson, Pharm.D.; Kelli Crowley, Pharm.D.; Sheila Hahner, MSN; Kelly Bricker, BSN; Celia Pulver, BSISM; Stacey Cote, MSN; Ann Terzis, MSN; Amanda Petrill, MSN; Tonya Evangelista, MSN; Susan Wible, MSN; Jacqueline Kwasniewski, BSN; Scott Coglio; Derek Whitehurst; Michael Hennigan.
